# The effect of external lateral stabilization on the use of foot placement to control mediolateral stability in walking and running

**DOI:** 10.7717/peerj.7939

**Published:** 2019-10-28

**Authors:** Mohammadreza Mahaki, Sjoerd M. Bruijn, Jaap H. van Dieën

**Affiliations:** 1 Faculty of Behavioural and Movement Sciences, VU University Amsterdam, Amsterdam, The Netherlands, Netherlands; 2 Faculty of Physical Education and Sport Sciences, Kharazmi University Tehran, Tehran, Iran; 3 Biomechanics Laboratory, Fujian Medical University, Quanzhou, Fujian, China

**Keywords:** Foot placement strategy, Balance, Gait stability, Walking, Running, Stepping strategy, External lateral stabilization

## Abstract

It is still unclear how humans control mediolateral (ML) stability in walking and even more so for running. Here, foot placement strategy as a main mechanism to control ML stability was compared between walking and running. Moreover, to verify the role of foot placement as a means to control ML stability in both modes of locomotion, this study investigated the effect of external lateral stabilization on foot placement control. Ten young adults participated in this study. Kinematic data of the trunk (T_6_) and feet were recorded during walking and running on a treadmill in normal and stabilized conditions. Correlation between ML trunk CoM state and subsequent ML foot placement, step width, and step width variability were assessed. Paired t-tests (either SPM1d or normal) were used to compare aforementioned parameters between normal walking and running. Two-way repeated measures ANOVAs (either SPM1d or normal) were used to test for effects of walking vs. running and of normal vs. stabilized condition. We found a stronger correlation between ML trunk CoM state and ML foot placement and significantly higher step width variability in walking than in running. The correlation between ML trunk CoM state and ML foot placement, step width, and step width variability were significantly decreased by external lateral stabilization in walking and running, and this reduction was stronger in walking than in running. We conclude that ML foot placement is coordinated to ML trunk CoM state to stabilize both walking and running and this coordination is stronger in walking than in running.

## Introduction

It is still unclear how humans walk and run with such ease, that is, stable and with low energy costs. Gait stability, i.e., maintaining a steady gait pattern without falling in the face of perturbations, requires control of the Center of Mass (CoM) relative to the Base of Support (BoS) (Arellano & Kram, 2011b; Bauby & Kuo, 2000; Kuo & Donelan, 2010). During walking and running, motions of the CoM relative to the BoS are thought to be controlled by passive dynamics as well as active processes (Arellano & Kram, 2011b; Bauby & Kuo, 2000; Kuo & Donelan, 2010). Small perturbations may be controlled by passive dynamics without Central Nervous System (CNS) involvement, and larger instabilities in the system are countered by active control, which requires sensing of perturbations, generating appropriate motor commands, and producing compensatory motions (Kuo & Donelan, 2010).

The foot placement strategy is the main mechanism to control medio-lateral (ML) stability in walking and running (Arellano & Kram, 2011a; Bruijn & Van Dieën, 2018; Donelan & Kram, 2001; Reimann, Fettrow & Jeka, 2018a; Seethapathi & Srinivasan, 2019). External lateral stabilization by means of a spring-like construction reduces ML CoM movement (Dean, Alexander & Kuo, 2007) and this coincided with a 24–60% reduction in step width in walking (Dean, Alexander & Kuo, 2007; Donelan et al., 2004; Ijmker et al., 2013) and 30–45% and 12.3% reductions in step width variability in walking (Donelan et al., 2004; Ijmker et al., 2013) and running (Arellano & Kram, 2011b), respectively. The coordination between CoM movements and step width is reciprocal, i.e., constraining CoM kinematics leads to adjustments of foot placement, but constraining foot placement also leads to adjustments of CoM kinematics (Arvin et al., 2016a; Arvin, Van Dieën & Bruijn, 2016b). This coordination between CoM displacement and foot placement is reflected in correlations of the CoM position and velocity during the swing phase with the subsequent foot placement (Hurt et al., 2010; Stimpson et al., 2018; Wang & Srinivasan, 2014). The active nature of the control of ML stability through foot placement is supported by studies on the effects of sensory illusions induced by vibration (Arvin et al., 2018), or visual perturbations (Reimann et al., 2018b) on this correlation, by studies that have related ML foot placement to swing phase muscle activity in control participants (Rankin, Buffo & Dean, 2014), and by studies that reported a weakened correlation in patients with neurological disorders (Dean & Kautz, 2015; Stimpson et al., 2019).

Although the foot placement strategy is important for control of gait stability, to date, we do not fully understand the mechanisms underlying the control of stability of walking and even less of running. It has been shown that humans run with step widths close to zero (Arellano & Kram, 2011a). A step width near zero may imply that there is a lower need for an accurate foot placement in running. In line with this, McClay and Cavanagh (McClay & Cavanagh, 1994) demonstrated that humans run by placing the foot along the middle of the body, which aligns the vertical ground reaction forces close to the CoM, minimizes the ML ground reaction forces on the body from step-to-step, and minimizes the moment generated about the AP axis (Cavanagh, 1987). Thus, most of the CoM displacement is directed forward, and ML motion is relatively small (Cavanagh, 1987). Decreasing ML CoM motion may be a strategy for control of stability during running, and if this is the case, the effect of external lateral stabilization on ML displacement of CoM, step width adjustment, and correlation of preceding ML CoM state with the subsequent ML foot placement (Wang & Srinivasan, 2014) will be lower in running than in walking. In the current study, we set out to test the idea that running is less dependent on foot placement to control ML stability than walking.

We hypothesized that (1) foot placement is coordinated with ML trunk CoM state in both walking and running, as reflected in a significant correlation between ML trunk CoM state during the swing phase and subsequent ML foot placement. (2) the foot placement strategy is more critical in walking than in running, as reflected in a significantly higher correlation between ML trunk CoM state and subsequent ML foot placement and a significantly greater step width and step width variability in walking compared to running. We further hypothesized that (3) external lateral stabilization decreases use of the foot placement strategy, as reflected by a significant reduction in the correlation between ML trunk CoM state and subsequent ML foot placement, alongside a significant decrease in step width, and step width variability. Since we expect more need for the foot placement strategy in walking than in running, we hypothesized that (4) the reduction in aforementioned parameters is significantly greater in walking than in running.^1^ 1Our initial research proposal for this project can be found at https://osf.io/mvkex/.^,^^2^ 2The effect of running speed on aforementioned parameters, as one of our pre-planned hypotheses, can be read in the Supplemental Information.

## Method

### Participants

After signing the informed consent, a convenience sample of 10 young (6 men, 4 women) participants (age: 27.70 ± 4.78 years, mass: 73.80 ± 8.57 kg, and height: 181.30 ± 6.57 cm) participated in this study, which had been approved by the local ethics committee of the Faculty of Behavioral and Movement Sciences of the Vrije Universiteit, Amsterdam (VCWE-2017-154). Exclusion criteria were: lower extremity injuries, history of surgery in the lower extremity, as well as any kind of impairments, medications, and infectious diseases which might affect walking mechanics or energy consumption. All of these exclusion criteria were self-reported by participants. Participants were asked to refrain from strenuous activity the day before experiments and to refrain from using coffee and alcohol on the day of the experiment.

### Experimental protocol

Participants visited the laboratory during one session and they were measured during walking and running on a motorized treadmill in two (normal, stabilized) conditions. The participants were familiarized with walking and running on the treadmill in each condition, and they were instructed not to resist the spring forces of the stabilization frame (Ijmker et al., 2013). Familiarization for each mode and each condition took about 2 min. Data collection started 10 min after the end of the familiarization protocol.

For each participant, first the conditions (normal and stabilized) were randomized and then speeds (walking at 1.25 m/s and running at 2.08, 2.50 and 2.92 m/s) were randomized within each condition. Participants completed 8 trials, each trial with a duration of 5 min. Trials were separated by a resting period of approximately 5 min.

### Experimental set-up

**Figure 1:**
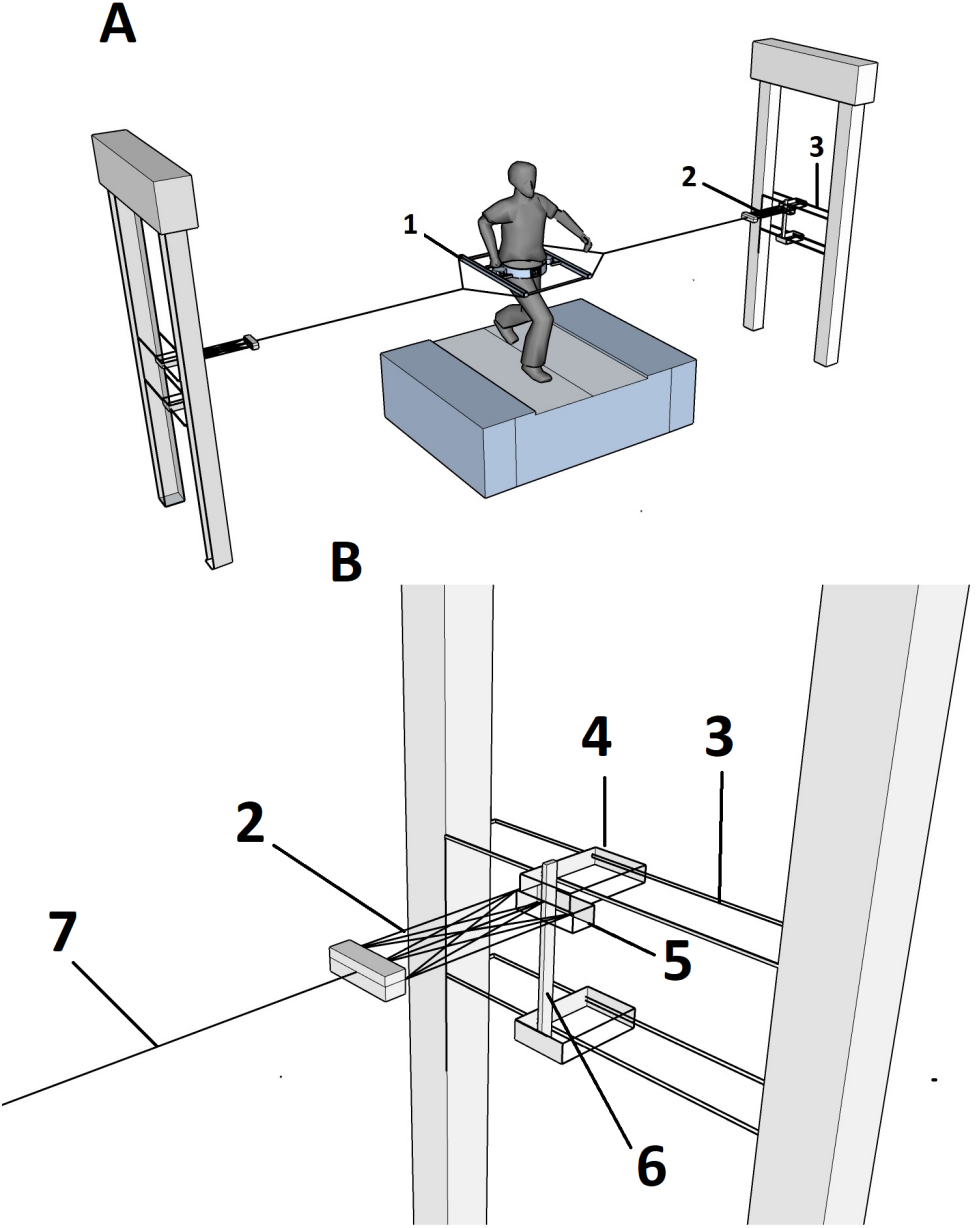
Schematic representation of the experimental set up. (A) Schematic representation of the experimental set up. Inset (B) shows the stabilization in more detail. (1) frame; (2) springs; (3) height-adjustable horizontal rail; (4) ball-bearing trolley freely moving in anterior-posterior direction; (5) slider freely moving in vertical direction; (6) vertical rail; and (7) rope attached to frame.

A light-weight frame (mass = 1.5 kg, see Fig. 1) was used for the external lateral stabilization condition, it was attached through a belt around the waist. Two sliders on both sides allowed participants to rotate their pelvis relative to the frame in the transverse plane, with minimal friction. Two stiff ropes attached to the frame on either side, joined each other at 0.5 m from the frame, providing space for free arm swing. From this junction, springs were attached to a slider on a vertical rail, which in turn was connected to two horizontal rails placed at the height of the pelvis of the participant. Thus, the set-up did not restrict movement in vertical and AP directions, nor rotations about the vertical axis, and transverse spring forces acted approximately at the level of the CoM during walking and running trials (Fig. 1). Springs with spring stiffness of approximately 1260 N/m were selected in this study since in a previous study no significant reductions of energy cost, step width, and step with variability were found beyond this stiffness (Ijmker et al., 2013).

### Instruments

Kinematic and kinetic data during walking and running trials were obtained from an Optotrak motion analysis system (Northern Digital Inc, Ontario, Canada), sampled at 100 samples/s and from force plates embedded in the treadmill (ForceLink b.v., Culemborg, the Netherlands), sampled at 1000 samples/s, respectively. Clusters of three infrared markers were attached to the thorax (over the T_6_ spinous process) and the heels.

### Data processing

All our data and codes used to process the data can be found at http://doi.org/10.5281/zenodo.3468501. Ground reaction force data were filtered with a 10 Hz cut-off frequency (2nd order, bidirectional Butterworth digital filter). Heel strike and toe off events were calculated from center of pressure data (Roerdink, Lamoth & Beek, 2008). Kinematic data from the Optotrak system were not filtered.

The trunk accounts for almost two-thirds of a person’s body mass and the effect of its motion on control of gait stability has been shown by a strong relationship between step-by- step variation in ML trunk CoM state and step width during walking (Hurt et al., 2010). The mean of the three infrared markers was used to approximate the ML trunk CoM position. The ML trunk CoM velocity was calculated as the first derivative of the ML trunk CoM position time-series. Each step was defined from toe off to heel strike (i.e., swing phase of gait cycle). Mid-stance was defined as 50% of the time between toe off and heel strike of the contralateral leg. While this may not coincide with the exact moment of mid-stance, it ensures that at this moment, we are absolutely certain that the foot is stationary, and thus the influence of erroneous detection of gait events is minimal. The ML position of the stance foot at mid-stance was defined as the origin and ML trunk CoM, and subsequent ML foot placement (position of the foot at the subsequent mid-stance) were expressed relative to this point. To further simplify the modeling (i.e., making sure that no offset was needed), all relevant variables (foot placement, ML trunk CoM, and ML trunk CoM velocity), were zero-centered by subtracting the mean for each percentage of the swing phase.

To investigate foot placement strategy in walking, previous studies have used a regression equation which predicts subsequent ML foot placement based on ML trunk CoM position and velocity at discrete time points (e.g., mid-swing (Arvin et al., 2018) or mid-stance (Hurt et al., 2010; Reimann et al., 2018b)) of the preceding swing phase. The *R*^2^ (i.e., the ratio of predicted foot placement variance to actual foot placement variance) has been reported as the primary outcome in previous studies (Arvin et al., 2018; Hurt et al., 2010; Wang & Srinivasan, 2014). *R*^2^ signifies the fit of regression equation which is between 0 to 100%. The higher *R*^2^ would represent a smaller difference between predicted and actual foot placements and thus would indicate a stronger correlation between ML trunk CoM state and subsequent ML foot placement (Hurt et al., 2010; Wang & Srinivasan, 2014). *R*^2^ higher than 50% has been interpreted as a high correlation between ML trunk CoM state and subsequent ML foot placement (Hurt et al., 2010). We used the following regression equation in which ML trunk CoM position and velocity time-series during swing phase predicted subsequent ML foot placement (Wang & Srinivasan, 2014):

\begin{eqnarray*}FP=\beta 1(i)\cdot \mathrm{CoM} \left( i \right) +\beta 2(i)\cdot \mathrm{V CoM} \left( i \right) +\varepsilon (i) \end{eqnarray*}

with *β*1 and *β*2 being the regression coefficients, ε the error, and *i* the indicator of the % of swing phase that was used for the prediction. Using ML trunk CoM state time-series during the preceding swing phase, the prediction of subsequent ML foot placement was repeated for each percentage of the swing phase. Therefore, our main outcome was the *R*^2^ time-series between predicted and actual foot placements.

**Figure 2:**
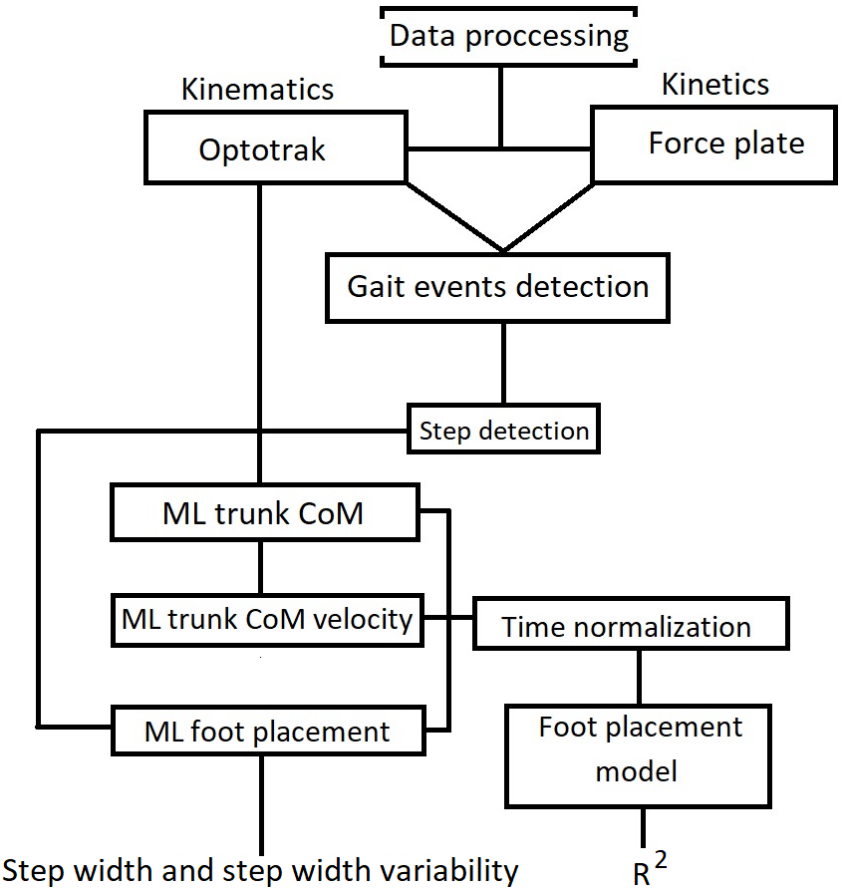
Flow of data processing adopted in this study.

Mean and variability of step width were calculated for each trial. Step width was defined as the mean of the distances between ML foot placement, and step width variability was defined as the standard deviation thereof. The procedure for data processing is illustrated in Fig. 2.

Energy costs were also measured during all conditions. Reduced energy costs in stabilized conditions would support that the control of ML stabilization requires energy consumption and differential effects between walking and running might indicate differences in these costs between these modes of locomotion. Since energy cost is not directly related to foot placement strategy, which is the main focus of this study, all the information about this parameter can be found in the Supplemental Information.

### Statistical analysis

Since our results indicated only very small differences between legs (see Fig. S1), we calculated the average *R*^2^ over legs. We selected walking at 1.25 m/s and running at 2.5 m/s, as a representative of running speeds, to test our hypotheses.^3^ 3Interested readers can run all analyses for each running speed by our provided codes.To test whether ML foot placement is coordinated with ML trunk CoM state in both walking at 1.25 m/s and running at 2.50 m/s, (hypotheses 1), the regression coefficients (*β*_1_ and *β*_2_) for each percentage of the swing phase in each individual participant were statistically tested by one sample t-tests. Significance of one or both of these regression coefficients would indicate a significant correlation between ML trunk CoM state and ML foot placement. To test whether this correlation was more pronounced in walking than running, (hypotheses 2), we tested for differences in *R*^2^, step width, and step width variability between normal walking at 1.25 m/s and running at 2.50 m/s, using a SPM (see below) paired *t*-test on the *R*^2^ time-series, and paired t-tests for step width and step width variability. Subsequently, we used repeated measures ANOVA (SPM-based for the *R*^2^ time-series, normal for step width and step width variability) with Condition and Locomotion mode as factors, to test for the effects of lateral stabilization (hypotheses 3), and we assessed the Condition X Locomotion mode interaction, to test for the differences in the effect of stabilization between walking at 1.25 m/s and running at 2.50 m/s, (hypotheses 4). The SPM analysis uses random field theory to identify regions in time-series that show significant effects (Pataky, Robinson & Vanrenterghem, 2013). This statistical approach captures features of the entire time-series, rather than a few discrete variables. The output of SPM provides an *t*-value (the second hypothesis) or F-value (the third and fourth hypotheses) for each sample of the R ^2^time-series, and a threshold corresponding to *α* set at 0.05. The values of t or F above the threshold indicate significant effects in the corresponding portion of the time-series.

## Results

**Figure 3:**
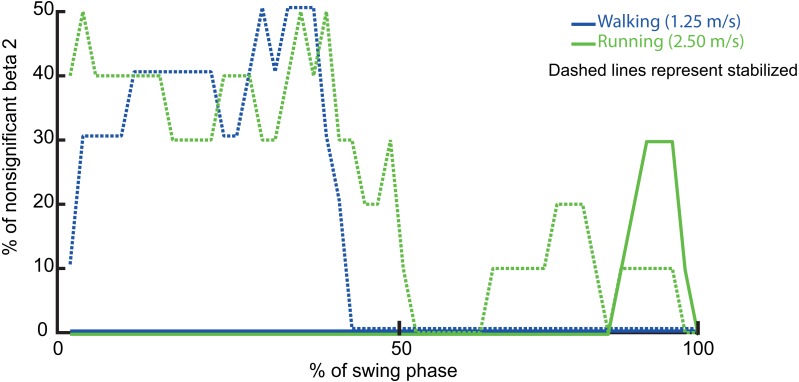
The % of nonsignificant β_2_’s during normal and stabilized conditions in walking and running trials per each % of swing phase.

**Figure 4:**
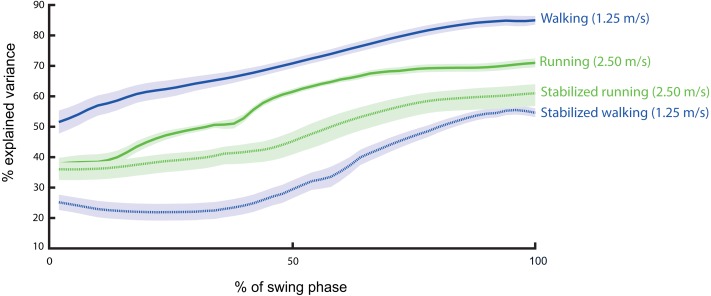
The ability of ML trunk CoM state to predict subsequent ML foot placement (*R*^2^) during normal (solid) and stabilized (dashed) conditions in walking (blue) and running (green). The shaded regions indicate standard error of *R*^2^.

The regression coefficients for ML trunk CoM position (*β*_1_) were significant for all regression equations and at all instants in the swing phase, while the regression coefficients for ML trunk CoM velocity (*β*_2_) were significant for most instances of the swing phase, with some exceptions. The percentage of nonsignificant *β*_2_-values was computed as the ratio of nonsignificant *β*_2_-values to the total number of *β*_2_-values multiplied by 100 for each percentage of the swing phase (Fig. 3). Thus, since one of the coefficients was significant for all regression equations, in line with our first hypothesis, the correlation between ML trunk CoM state and subsequent ML foot placement was significant during both walking and running. The *R*^2^ values were high, ranging between ∼0.52–0.85 from 0–100% of the swing phase in walking and between ∼0.50–0.71 from 35–100% of the swing phase in running (Fig. 4).

**Figure 5:**
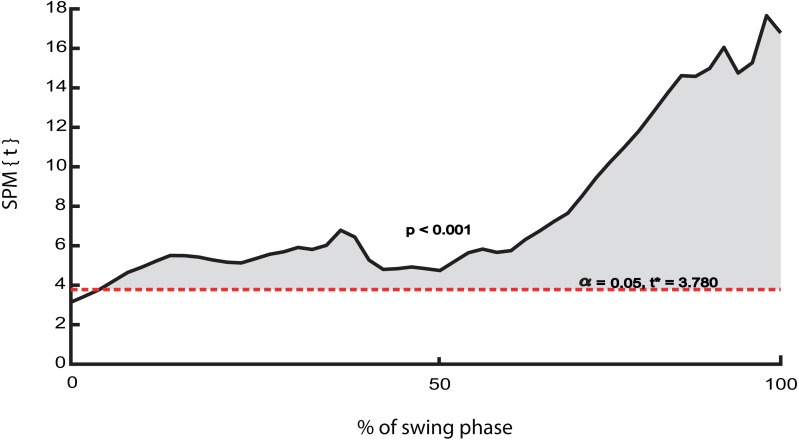
The differences of *R*^2^ between normal walking and running. The shaded areas indicate significant effects in the corresponding portion of the swing phase (based on the results of SPM paired t-test).

**Figure 6:**
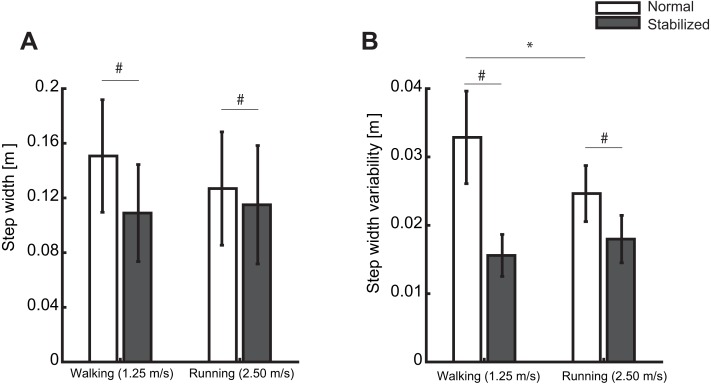
The effect of external lateral stabilization on (A) step width and (B) step width variability. Condition effect: The effect of external lateral stabilization on (A) step width and (B) step width variability in walking and running. # represents the significant differences of step width and step width variability between normal and stabilized conditions (based on the results of Bonferroni post-hoc tests). * represents the significant differences of step width and step width variability between normal walking and running (based on the results of paired t-test). The error bars represent the standard deviation.

In line with our second hypothesis, we found a significantly stronger correlation between ML trunk CoM state and subsequent ML foot placement in walking than in running from 0–100% of the swing phase (Figs. 4 and 5), as well as a significantly greater step width variability (*t*(1, 9) = 4.17, *p* = 0.002) in walking than running, however the differences of step width was not significant (*t*(1, 9) = 2.21, *p* = 0.05) (Figs. 6A and 6B).

**Figure 7:**
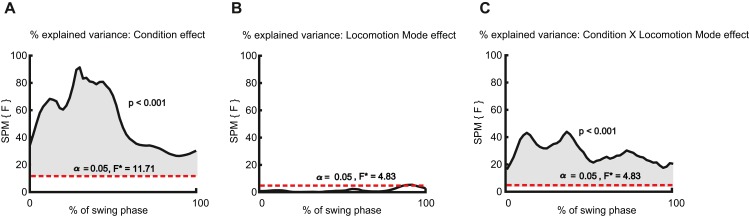
The effect of lateral stabilization on *R*^2^ in walking and running. (A) Condition effect: The effect of external lateral stabilization on *R*^2^ in walking and running. (B) Locomotion mode effect: The differences of *R*^2^ between walking and running in both conditions (normal & stabilized). (C) Interaction effect (condition × locomotion mode effect): The differences of external lateral stabilization effect on *R*^2^ between walking and running. The shaded areas indicate significant effects in the corresponding portion of the swing phase.

In line with our third hypothesis, external lateral stabilization significantly decreased *R*^2^ to ∼0.25–0.55 and ∼0.36–61% during 0–100% of the swing phase in walking and running, respectively (Figs. 4 and 7A). External lateral stabilization also significantly decreased step width (Condition effect; *F*(1, 9) = 32.49, *p* ≤ 0.001, and step width variability (Condition effect; *F*(1, 9) = 100.24, *p* ≤ 0.001) (Figs. 6A and 6B).

In line with our fourth hypothesis, the effect of external lateral stabilization on *R*^2^ was larger in walking than in running (Condition X Locomotion mode effect, Fig. 7C). In addition, the effect of external lateral stabilization on step width and step width variability was larger in walking than in running (Condition X Locomotion mode effect, (Figs. 6A and 6B) (*F*(1, 9) = 15.63, *p* = 0.003 for step width and *F*(1, 9) = 23.21, *p* < 0.001 for step width variability).

## Discussion

Our results demonstrated a strong correlation between ML trunk CoM state in the swing phase of the gait cycle and subsequent ML foot placement during both walking and running. ML trunk CoM state explained over 50% of the variance in ML foot placement during the entire swing phase in walking and the last 65% of swing phase in running, respectively. Our hypothesis that the foot placement strategy is more critical in walking than in running, was supported by a stronger correlation between ML trunk CoM state during the swing phase and subsequent ML foot placement, as well as greater step width variability in walking than in running. Furthermore, our hypothesis that external lateral stabilization significantly decreases the correlation of ML foot placement to ML trunk CoM state, was also supported for both modes of locomotion. This hypothesis was also supported by significant reduction in step width and step width variability in the stabilized condition compared to the normal condition. The hypothesis that the foot placement strategy is more critical in walking than in running was supported by stronger reductions in the correlation between ML trunk CoM state and subsequent ML foot placement, and in step width, and step width variability in stabilized walking than in stabilized running.

Our results confirmed that ML foot placement is coordinated to ML trunk CoM state in walking. Similar to previous studies, which reported that 50–84% of ML foot placement variance can be explained by ML trunk, ML pelvis, or ML whole-body CoM state during walking (Hurt et al., 2010; Stimpson et al., 2018; Wang & Srinivasan, 2014), our results indicated high predictive ability of ML trunk CoM state on subsequent ML foot placement, with *R*^2^ ranging between 52–85% during the entire swing phase in walking. Recently, Seethapathi & Srinivasan (2019) reported that ML foot placements relative to CoM position are predicted by mid-swing ML CoM velocity in running, with *R*^2^ values ranging 62–64%. Similarly, our results indicated a high correlation between ML trunk CoM state and subsequent ML foot placement (*R*^2^ = 50–71%) during the last 65% of the swing phase in running. The high predictive ability of ML trunk CoM state in walking and running could be due to active control of ML stability through foot placement, and could also be due to passive dynamic coupling of lower extremity movements to movements of the upper body. Although the results of current study cannot answer the question whether active control or passive coupling is the underlying cause of this correlation, active control of ML stability through foot placement is supported by studies on the effects of sensory illusions induced by vibration (Arvin et al., 2018), or visual perturbations (Reimann et al., 2018b) on this correlation, and by studies that have related ML foot placement to swing phase muscle activity (Rankin, Buffo & Dean, 2014). On the other hand, we cannot rule out that the passive dynamics play a role in the correlation between ML trunk CoM state and subsequent ML foot placement that we report. Thus, further studies are needed to elucidate the degree to which active control contributes to foot placement coordination in walking and running.

Our results indicated that the correlation between ML trunk CoM state and subsequent ML foot placement is less strong in running than in walking. It has been suggested that the foot placement strategy begins earlier in *walking* when less time is available to complete the step (i.e., during walking at higher speeds) (Rankin, Buffo & Dean, 2014; Stimpson et al., 2018). However, the more pronounced reduction in step duration in running could limit the possibility of using foot placement strategy. If this is the case, one step after a deviation of ML trunk CoM state might not be enough to restore ML stability, and more consecutive steps might be required to stabilize ML trunk CoM state in running. However, using Goal Equivalent Manifold framework, It has been reported that humans correct stride-to-stride variability both more quickly and more directly in running than in walking (Dingwell, Bohnsack-McLagan & Cusumano, 2018). Such a tighter control in running might result from other stability strategies, rather than foot placement strategy. For instance, during running an absorption strategy, allowed by flexion in the lower limb, during the stance phase may be used to control the ML trunk CoM state, which may limit the need for accurate foot placement (similar as the impulse control proposed by Seethapathi & Srinivasan, 2019).

It has been reported that external lateral stabilization decreases ML displacement of the CoM (Dean, Alexander & Kuo, 2007), accompanied by a 24–60% reduction in step width in walking (Dean, Alexander & Kuo, 2007; Donelan et al., 2004; Ijmker et al., 2013) and 30–45% in step width variability in walking (Donelan et al., 2004; Ijmker et al., 2013). Our results indicate that external lateral stabilization decreased the correlation between ML trunk CoM state and subsequent ML foot placement, alongside a reduction in step width and step width variability during stabilized walking. The results of the current study also indicate that external lateral stabilization decreases the correlation between ML trunk CoM state and subsequent ML foot placement, step width, and step width variability in running, although less so than for walking, in line with a smaller decrease in step width variability of about 12% with external stabilization reported previously (Arellano & Kram, 2011b). This smaller decrease may suggest that subjects need more foot placement strategy during stabilized running than during stabilized walking. This would appear to contradict the notion that the foot placement strategy is less important during normal running than normal walking. However, there may be several alternative explanations. First of all, the external lateral stabilization may have different effects on ML stability in running and walking; it may be less effective during running, as the ML forces may affect body movements differently during the flight phase in running compared to the single leg stance phase in walking. In single leg stance, the spring forces and ground reaction forces on the stance leg may produce a rotational couple, which does not occur during the flight phase in running. It could be that this rotational component is key to stabilizing subjects. Thus, the stabilizing effect may be different between walking and running, but for now, this remains speculation. A second explanation, may be that subjects do not experience the frame as sufficiently stabilizing in running and thus do not “offload” control to the frame as much as they do in walking. However, participants were familiarized with all conditions, and did not express feelings of discomfort during any of the conditions, rendering this unlikely.

## Conclusion

ML trunk CoM state explained over 50% of the variance in ML foot placement during the entire swing phase in walking, and the last 65% of swing phase in running. This suggests that ML foot placement is correlated to ML trunk CoM state to actively control ML stability at the end of gait cycle in walking and running. Still the passive dynamics coupling between ML trunk CoM movement and ML foot placement might play role on this correlation. The foot placement strategy appears more critical in walking than in running, as the correlation between ML trunk CoM state and subsequent ML foot placement was higher in walking than running. External lateral stabilization decreased this correlation, step width, and step width variability in both walking and running, with stronger reductions during the former. This may imply that there is a higher need for an accurately coordinated foot placement in walking.

##  Supplemental Information

10.7717/peerj.7939/supp-1 Figure S1 (A) % of variance in ML foot placement that can be explained by ML trunk CoM state (*R*^2^) in walking and running. (B) The differences of *R*^2^ between left and right legs in walking and running

10.7717/peerj.7939/supp-2 Figure S2 The effect of speed on *R*^2^ in running (A) % of variance in ML foot placement that can be explained by ML trunk CoM state (*R*^2^) in running with three different speeds [2.08, 2.50, and 2.92 m/s]. (B) The effect of running speeds (2.08, 2.50, and 2.92 m/s) on *R*^2^. The shaded regions indicate standard error of *R*^2^.

10.7717/peerj.7939/supp-3 Figure S3 Effect of speed on step width in running Step width was significantly decreased by increasing in running speed (*F*(1, 2) = 9.25, *p* = 0.002).

10.7717/peerj.7939/supp-4 Figure S4 Effect of speed on step width variability in running There was no significant main effect of speed on step width variability in running (*F*(1, 2) = 1.48, *p* = 0.254).

10.7717/peerj.7939/supp-5 Figure S5 The effect of lateral stabilization on energy cost in walking and running. Condition effect: The effect of external lateral stabilization on energy cost in walking and running. # represents the significant differences of energy cost between normal and stabilized conditions (based on the results of Bonferroni post-hoc). Error bars represent standard deviation.

10.7717/peerj.7939/supp-6 Supplemental Information 1 Raw data applied for data analyses and preparation for Fig. S1–S6

## Additional Information and Declarations

### Data Availability

The following information was supplied regarding data availability: The data and codes are available at Zenodo: Mohammadreza Mahaki, Sjoerd M. Bruijn, & Jaap H. van Dieën. (2019). The effect of external lateral stabilization on the use of foot placement to control mediolateral stability in walking and running [Data set]. PeerJ. Zenodo. http://doi.org/10.5281/zenodo.3468501.
