# Supplementary material for: The effect of external lateral stabilization on the use of foot placement to control mediolateral stability in walking and running
Source: PeerJ. 2019 Oct 28;7:e7939. doi: 10.7717/peerj.7939 (PMC6822599; doi:10.7717/peerj.7939)
Supplement: Supplemental Information 1 [file peerj-07-7939-s006.docx]

# The differences between legs in walking and running

## % of variance in ML foot placement that can be explained by ML trunk CoM state (R^2^)

Very small, nonsignificant differences were found between right and left legs for R^2^ during swing phases of walking and running (**Fig. S1**).


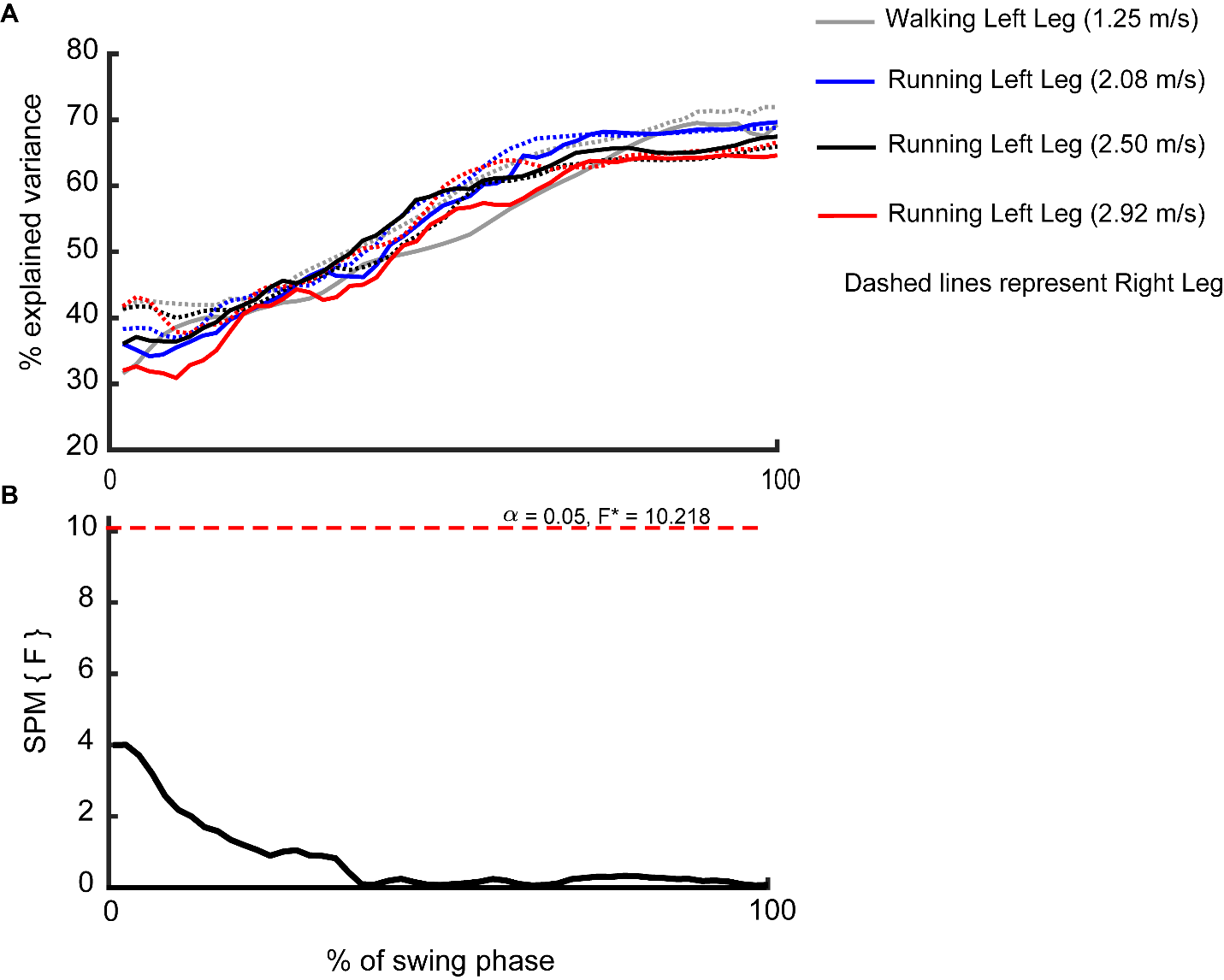


**Fig. S1. (A)** % of variance in ML foot placement that can be explained by ML trunk CoM state (R^2^) in walking and running. **(B)** The differences of R^2^ between left and right legs in walking and running.

We calculated the average of R^2^ over legs in walking and running because our results indicated very small, nonsignificant differences between legs during swing phase (**Fig. S1**). Then, the effect of running speed on this parameter was considered:

# The effect of running speeds (2.08, 2.50, and 2.92 m/s) on R^2^, step width, and step with variability:

The effect of speed on the stability of walking has been investigated in several studies [1-4], however there is a lack of information on running. Most relevant for our present focus, Wang and Sirinivasan [5, 6] reported that the correlation between ML CoM state and ML foot placement was not influenced by walking speeds between 1.0, 1.2, and 1.4 m/s. In agreement with these results, Stimpson et al. [5] reported that this correlation was not influenced by walking speeds between 1.0 and 1.2 m/s, but it was affected by speeds between 0.2 – 1.0 m/s, with less strong correlation at lower speeds. In this study, we intended to test the idea that speed influences coordination between ML trunk CoM state and subsequent ML foot placement, step width, and step width variability in running.

### Statistical analysis

we used one-way repeated measures ANOVA (SPM-based for the R^2^ time-series, normal for step width and step width variability) to test the main effect of speed with three levels (2.08, 2.50, and 2.92 m/s) on these parameters.

### Results

## R^2^

Very small, nonsignificant differences of R^2^ were found between different running speeds (**Fig. S2**).


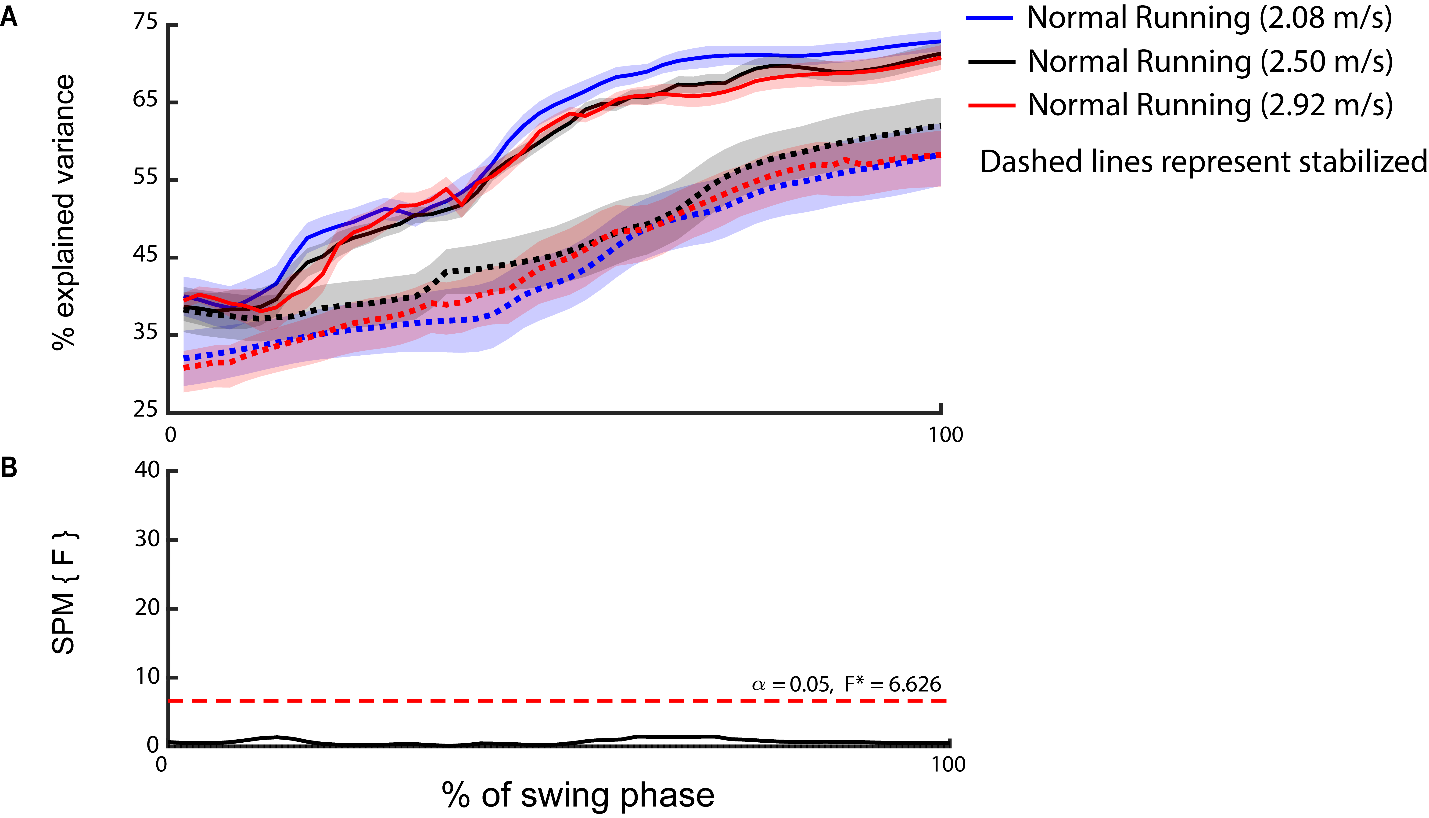


**Fig. S2. (A)** % of variance in ML foot placement that can be explained by ML trunk CoM state (R^2^) in running with three different speeds [2.08, 2.50, and 2.92 m/s]. **(B)** The effect of running speeds (2.08, 2.50, and 2.92 m/s) on R^2^. The shaded regions indicate standard error of R^2^.

## Step width

Step width was significantly decreased by increasing in running speed (*F (1, 2) = 9.25,* *p = 0.002*) (**Fig. S3**).


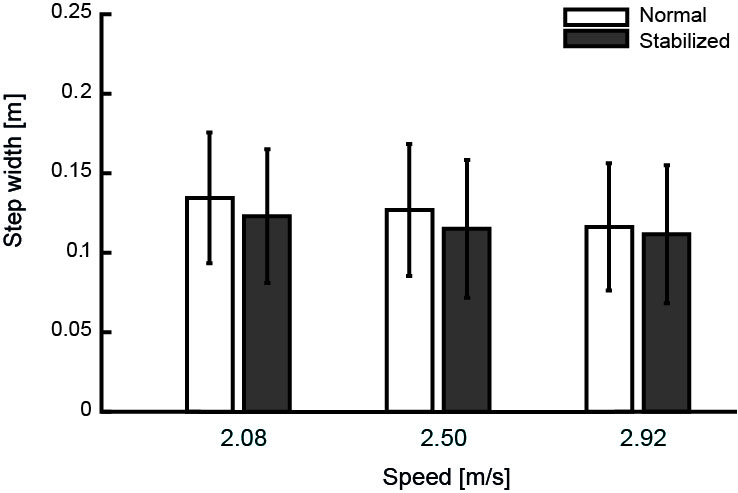


**Fig. S3.** Effect of speed on step width in running.

## Step width variability

There was no significant main effect of speed on step width variability in running (*F (1, 2) = 1.48,* *p = 0.254*) (**Fig. S4**).


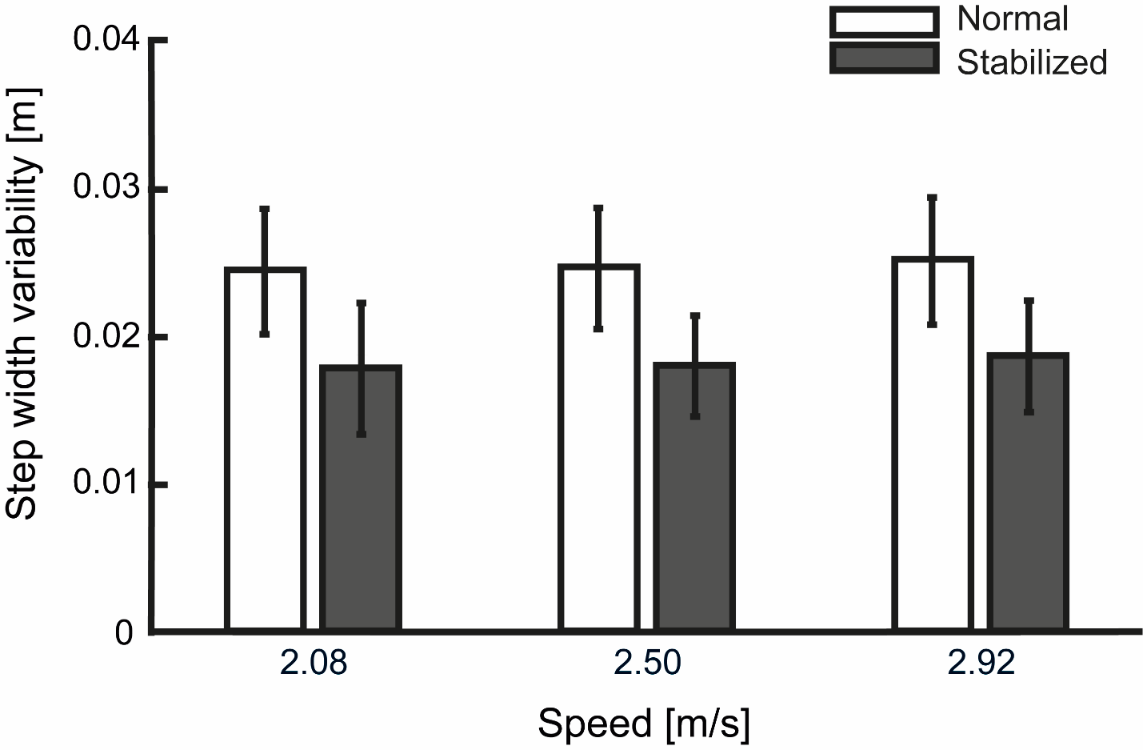


**Fig. S4**. Effect of speed on step width variability in running.

### Discussion

It has been reported that the foot placement strategy in walking, which is reflected by a correlation between the ML CoM state during the swing phase and the subsequent ML foot placement, is not affected by walking speed between 1.0-1.4 m/s [5, 6]. We extended this to running and our results showed that the foot placement strategy in running is not affected by speeds ranging between 2.08-2.92 m/s.

## Energy cost

Energy costs of ML stability control in walking and running was also investigated. Reduced energy costs in stabilized conditions would support that the control of ML stabilization requires energy consumption[7-10], and differential effects between walking and running might indicate differences in these costs between these modes of locomotion. Since energy cost is not directly related to foot placement strategy, which is the main focus of this study, all the information about this parameter can be read below:

### Instruments

Breath-by-breath oxygen consumption was also obtained from a pulmonary gas exchange system (Cosmed K4b^2^, Cosmed, Italy).

### Data processing

Oxygen uptake ($\dot{V}O_{2}$; ml min^-1^) and respiratory exchange ratio (RER) were determined with the pulmonary gas exchange system during the last minute of each trial. The metabolic rate reached a plateau within the 5- minutes trial, as was confirmed through visual inspection. We calculated gross metabolic rate (E_gross_; J kg^-1^ min^-1^) as [11]:

$\mathbf{E}_{\mathbf{gross}}\boldsymbol{=((4.940\cdot RER+16.40)\cdot}\dot{\mathbf{V}}\mathbf{O}_{\mathbf{2}}\boldsymbol{)}$**/body mass (kg)**

Resting metabolic rate, determined with the same method as we did for gross metabolic rate during seated position for 5 min prior to the trials, was subtracted from gross metabolic rate to calculate net metabolic rate during walking and running. To calculate net energy cost (EC; J kg^-1^ m^-1^), net metabolic rate was divided by speed (m min^-1^).

### Statistical analysis

Running at 2.50 m/s was selected as a representative of running speeds. We were only interested in the effects of external lateral stabilization on energetic cost in walking at 1.25 m/s and running at 2.50 m/s, hypothesis 3, and the differences of external lateral stabilization effect on energetic cost between walking at 1.25 m/s and running at 2.50 m/s, hypothesis 4. To test these hypotheses, a two-way repeated analysis of variance with conditions (normal vs stabilized) and mode of locomotion (walking at 1.25 m/s vs running at 2.50 m/s) as within-subject factors was conducted to evaluate the effect of external lateral stabilization and interaction (mode of locomotion X condition) on energy cost^[[1]](#footnote-1)^.

### Results

In contrast with expectations, energy costs were significantly higher in the stabilized conditions (Condition effect; *F (1, 9) = 5.26, p = 0.047*), but the interaction effect was not significant (Interaction effect*; F (1, 9) = 4.53, p = 0.062*).(**Fig. S5**).


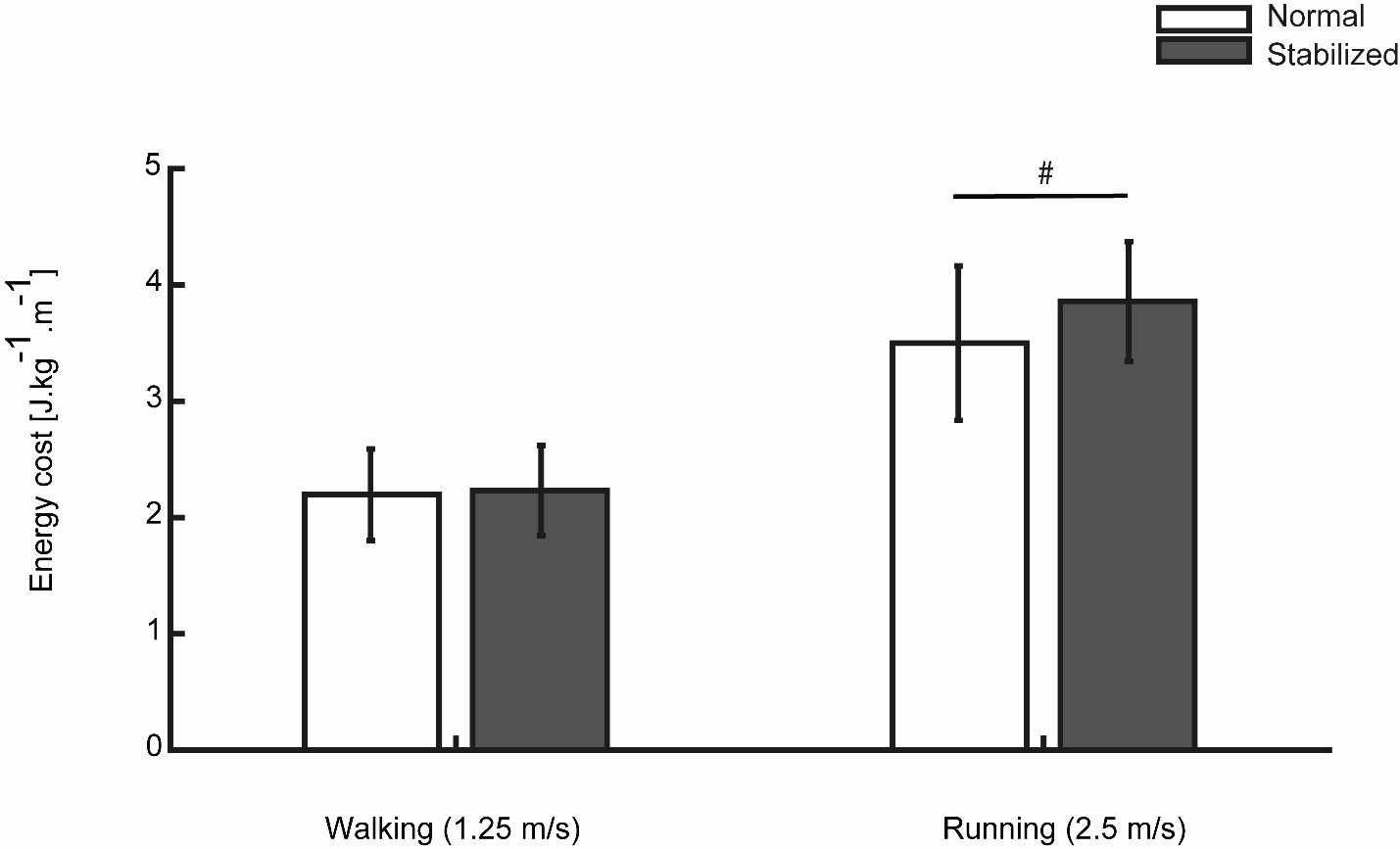


**Fig. S5.** Condition effect: The effect of external lateral stabilization on energy cost in walking and running. # represents the significant differences of energy cost between normal and stabilized conditions (based on the results of Bonferroni post-hoc). Error bars represent standard deviation.

### Discussion

We measured energy to assess costs of stability control. Reduced energy costs in stabilized walking, as has been reported before [7-10] would support that ML stabilization is an active process and differential effects between walking and running might indicate differences in these costs between these modes of locomotion. Previous studies reported mixed results on the effects of external lateral stabilization on energy costs. Several studies reported significant reductions in energetic cost due to stabilization. Donelan et al. [8] reported a significant 5.7% and 9.2% reduction in EC during preferred and zero step width conditions respectively, while walking with arm swing restriction. Ortega et al. [10] also reported significant effects of lateral stabilization on EC in walking in which participants were allowed familiarization, walked with preferred step width, and participants were allowed normal arm swing. With arm swing the effect of lateral stabilization was slightly lower (a significant 3-4% reduction), compared to walking without arm swing (a significant 6-7% reduction). Arellano et al. reported significant 5.5% and 2% reductions in EC while walking and running, respectively, without arm swing at a zero target step width [12]. In contrast, Dean et al. [7] did not find a significant reduction in EC during walking with stabilization at preferred step width, although they did find an effect in a prescribed zero step width condition. IJmker et al. [9] reported a significant reduction in EC during walking with stabilization, but only after removal of an outlier. In a second study by IJmker et al. [13] with able-bodied participants and people with a lower limb prosthesis, the effect of stabilization failed to reach significance. While it was speculated that the lateral stabilization might impede functional medio-lateral motion in amputees, no satisfying explanation was provided for the lack of effect in the control group. The reported significant reduction of EC during the stabilized condition can be attributed to the EC of controlling frontal plane gait stability if the bilateral spring forces act only mediolaterally on body CoM (i.e. provide stability just in mediolateral direction). For this, bilateral springs need to be connected to a height-adjustable horizontal trolley which can move freely and in-phase with the body CoM in the anterior-posterior direction. Fixed springs in anterior-posterior direction, used in previous studies, may add anterior-posterior forces induced by the springs and as such may provide assistance or resistance. This potential effect expected to be larger when shorter ropes are used to connect the springs to the subject and may by providing assistance have increased some of the EC savings of external lateral stabilization. We did not find a decrease in energetic cost during stabilized walking. Instead, energy costs slightly increased, especially during running. This appears to contradict the notion that ML stabilization is an active process which entails energy cost. However, there may be several explanations. Firstly, unintended effects of the external stabilization, e.g. on propulsion may have outweighed the benefits. Secondly, we may not have had long enough habituation time to allow for full familiarization. Lastly, differences between our set up and the set up used in other studies may have caused an unwanted increase in energetic cost; for instance, we allowed vertical and transverse motions (by adding sliders in those directions). To investigate whether external lateral stabilization reduces the energy cost during walking and in which conditions, further analyses such as meta-regression could to be performed on data from the studies mentioned. Our results showed that foot placement is used to control ML stability in walking and running. The energy cost of this strategy appears to be low, as the decrease in the use of the foot placement strategy during stabilized walking and running did not lead to decreases in energy costs. Low energy costs may explain why foot placement is likely to be preferred over other stability control strategies, such as control through ankle moments [14-16].

# References

1. Bruijn, S.M., J.H. van Dieën, O.G. Meijer, and P.J. Beek, *Is slow walking more stable?* Journal of biomechanics, 2009. **42**(10): p. 1506-1512.

2. Dingwell, J.B. and L.C. Marin, *Kinematic variability and local dynamic stability of upper body motions when walking at different speeds.* Journal of biomechanics, 2006. **39**(3): p. 444-452.

3. England, S.A. and K.P. Granata, *The influence of gait speed on local dynamic stability of walking.* Gait & posture, 2007. **25**(2): p. 172-178.

4. Hak, L., H. Houdijk, F. Steenbrink, A. Mert, P. van der Wurff, P.J. Beek, and J.H. van Dieën, *Speeding up or slowing down?: Gait adaptations to preserve gait stability in response to balance perturbations.* Gait & posture, 2012. **36**(2): p. 260-264.

5. Stimpson, K.H., L.N. Heitkamp, J.S. Horne, and J.C. Dean, *Effects of walking speed on the step-by-step control of step width.* Journal of biomechanics, 2018. **68**: p. 78-83.

6. Wang, Y. and M. Srinivasan, *Stepping in the direction of the fall: the next foot placement can be predicted from current upper body state in steady-state walking.* Biology letters, 2014. **10**(9): p. 20140405.

7. Dean, J.C., N.B. Alexander, and A.D. Kuo, *The effect of lateral stabilization on walking in young and old adults.* IEEE Transactions on Biomedical Engineering, 2007. **54**(11): p. 1919-1926.

8. Donelan, J.M., D.W. Shipman, R. Kram, and A.D. Kuo, *Mechanical and metabolic requirements for active lateral stabilization in human walking.* Journal of biomechanics, 2004. **37**(6): p. 827-835.

9. Ijmker, T., H. Houdijk, C.J. Lamoth, P.J. Beek, and L.H. van der Woude, *Energy cost of balance control during walking decreases with external stabilizer stiffness independent of walking speed.* Journal of biomechanics, 2013. **46**(13): p. 2109-2114.

10. Ortega, J.D., L.A. Fehlman, and C.T. Farley, *Effects of aging and arm swing on the metabolic cost of stability in human walking.* Journal of biomechanics, 2008. **41**(16): p. 3303-3308.

11. Garby, L. and A. Astrup, *The relationship between the respiratory quotient and the energy equivalent of oxygen during simultaneous glucose and lipid oxidation and lipogenesis.* Acta Physiologica Scandinavica, 1987. **129**(3): p. 443-444.

12. Arellano, C.J. and R. Kram, *The energetic cost of maintaining lateral balance during human running.* Journal of Applied Physiology, 2011. **112**(3): p. 427-434.

13. IJmker, T., S. Noten, C. Lamoth, P. Beek, L. van der Woude, and H. Houdijk, *Can external lateral stabilization reduce the energy cost of walking in persons with a lower limb amputation?* Gait & posture, 2014. **40**(4): p. 616-621.

14. Bruijn, S.M. and J.H. van Dieën, *Control of human gait stability through foot placement.* Journal of The Royal Society Interface, 2018. **15**(143): p. 20170816.

15. Hof, A. and J. Duysens, *Responses of human ankle muscles to mediolateral balance perturbations during walking.* Human movement science, 2018. **57**: p. 69-82.

16. Reimann, H., T. Fettrow, and J.J. Jeka, *Strategies for the control of balance during locomotion.* Kinesiology Review, 2018. **7**(1): p. 18-25.

1. Our initial research proposal for this project can be found at <https://osf.io/mvkex/>. [↑](#footnote-ref-1)
